# Fournier’s Gangrene in a Female Diabetic Patient: A Case Report

**DOI:** 10.7759/cureus.21293

**Published:** 2022-01-16

**Authors:** Enkhmaa Luvsannyam, Sataj Johnson, Veronica Velez, Archana Bottu, Tasanee Rungteranoont, Megan A Hammersla, Frederick Tiesenga

**Affiliations:** 1 Surgery, California Institute of Behavioral Neurosciences & Psychology, Fairfield, USA; 2 Medicine, California Institute of Behavioral Neurosciences & Psychology, Fairfield, USA; 3 General Surgery, West Suburban Medical Center, Chicago, USA

**Keywords:** obesity, diabetes, soft tissue infection, fournier’s gangrene, necrotizing fasciitis

## Abstract

Necrotizing fasciitis is a rare but potentially fatal deep infection involving subcutaneous tissue and fascia. The infection can occur in all parts of the body and can cause acute onset pain, swelling, fever, malaise, and tachycardia with or without evidence of skin inflammation. Risk factors include recent surgery, diabetes, trauma, intravenous drug use, alcoholism, and chronic illnesses. This case involves a 35-year-old female with a past medical history of hypertension, type II diabetes mellitus, and obesity presenting with a painful vulvar lump, which progressed rapidly into extensive necrotizing soft tissue infection despite the incision and drainage of the vulvar abscess, marsupialization, and antibiotic therapy. The patient underwent multiple surgical debridements with intense medical treatment and wound vacuum-assisted closure therapy. Uncontrolled diabetes and obesity significantly increase the risk of necrotizing fasciitis. Fournier’s gangrene should be suspected in patients with comorbid conditions and a presentation of a urogenital abscess. This case highlights the importance of prompt diagnosis and treatment of necrotizing fasciitis in a timely manner.

## Introduction

Commonly known by the frightening term, “flesh-eating disease,” necrotizing fasciitis is a rare but potentially fatal deep infection involving subcutaneous tissue and fascia. It can occur in all parts of the body and can be caused by a mixed infection of anaerobic and aerobic bacteria. Risk factors include recent surgery, diabetes, trauma, intravenous drug use, alcoholism, and chronic illnesses. Features of necrotizing fasciitis include acute onset pain, swelling, fever, malaise, and tachycardia with or without evidence of skin inflammation [1].

In 1883, Jean Alfred Fournier, a French physician who studied venereal disease, described an idiopathic rapidly progressive gangrene with acute onset in healthy young men, naming it Fournier’s gangrene [2]. Modern medicine and improvements in pathophysiology over time have considered Fournier’s gangrene to no longer be idiopathic and limited to young males, but Fournier’s clinical description stayed accurate [2]. Fournier’s gangrene is a form of necrotizing fasciitis localized to the external genitalia and perineum. It is described as a life-threatening urologic emergency characterized by progressive necrotizing infection, and it makes up 21% of necrotizing fasciitis cases [3]. A previous case series described an overall incidence of 1.6 cases of Fournier’s gangrene per 100,000 males per year, consisting of less than 0.02% of hospital admissions, meaning that Fournier’s gangrene is rarest in females [3]. Using ICD diagnosis codes for gangrene, vulvovaginal gland abscess, or vulvar abscess, out of the 15.1 million females in the data set of that case series, 39 females had an accurate diagnosis of Fournier’s gangrene [3]. Female data and statistics are still limited today.

Diabetes mellitus (DM) is a common comorbidity in Fournier’s gangrene with a higher risk of mortality and longer hospital stays in these patients due to the microvasculature of many soft tissues and organs being affected [4,5]. Since the focus of infection is the genitourinary tract, Fournier’s gangrene can spread rapidly. If not treated immediately, sepsis, toxic shock syndrome, and multi-organ failure may occur, allowing mortality to approach 100%. Diagnosis consists of clinical examination, imaging that may show gas in the soft tissue, and tissue cultures to determine organism involvement. Treatment consists of early surgical exploration and debridement, broad-spectrum antibiotic coverage, and hemodynamic stabilization [5].

## Case presentation

**Figure 1:**
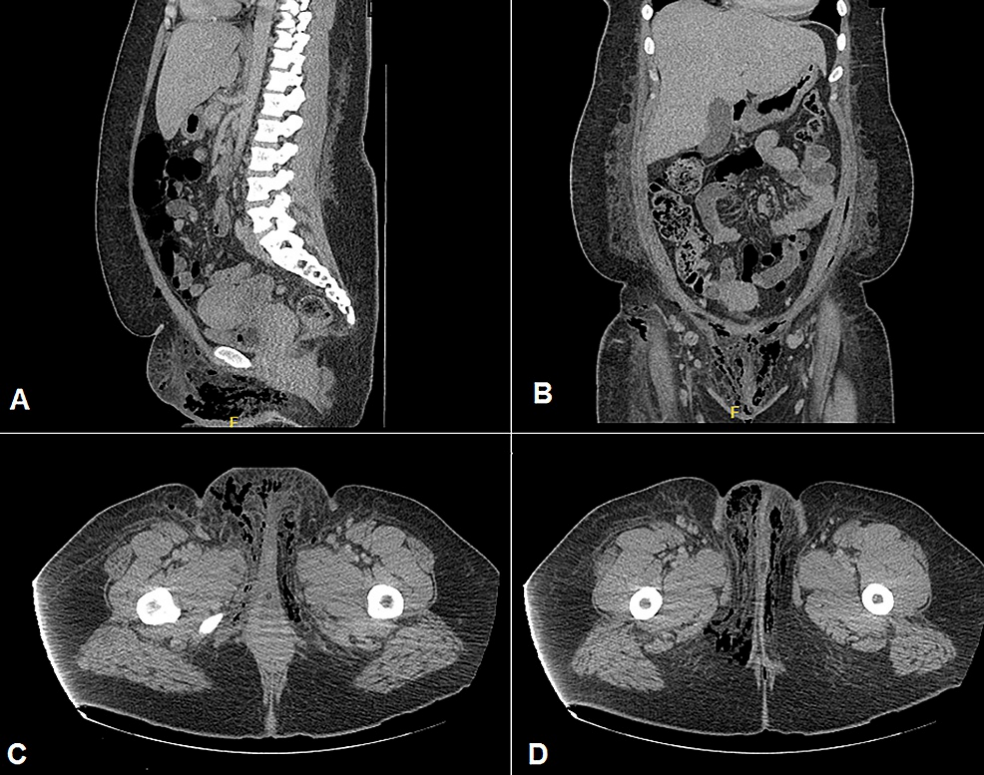
CT of abdomen and pelvis with contrast demonstrating subcutaneous emphysema in the lower abdominal and pubic region in (A) sagittal and (B) coronal sections; in bilateral perineum, labia and groin region in axial sections (C, D).

A 35-year-old female with a past medical history of hypertension, insulin-dependent type II DM, and obesity presented to the emergency department (ED) in Chicago, the United States (US), complaining of a vulvar lump associated with fever. In the ED, she was prescribed trimethoprim/sulfamethoxazole for vulvar abscess. Despite antibiotic treatment for seven days, the patient returned to the ED due to worsening symptoms of vulvar pain, fever, and chills. Physical examination revealed enlarged bilateral vulvar lumps with erythema and induration. The patient was admitted and had an incision and drainage of the vulvar abscess and marsupialization the following morning. On postoperative day 1, the patient remained febrile and tachycardic despite antibiotics and Tylenol administration. The patient was complaining of worsening pain and fullness around her wound site. Her postoperative white blood cell (WBC) count was 40 x 10^9^/L, glucose remained at 300-400 mg/dL, and HbA1c was 9.0. Examination revealed new firm nodules on bilateral perilabial areas with cellulitis extending to her suprapubic area and groin. Her abdomen was distended and tender to palpation in the lower quadrants with rebound tenderness. An emergent abdominopelvic computed tomography (CT) demonstrated extensive subcutaneous emphysema of the perineum extending into the labia and groin bilaterally, as well as lower anterior abdominal wall highly suspicious for Fournier’s gangrene (Figures 1A-1D).

The patient was taken to the operating room immediately for a wide excisional debridement of perilabial abscess and necrotizing fasciitis. Intra-operatively, large amounts of purulent fluid and extensive necrotic tissue were found in the bilateral perilabial, perineum, and lower abdominal wall region. A large amount of skin, subcutaneous tissue, and fascia were excised due to widespread necrotic tissue leaving a 32 x19 x 7 cm full-thickness wound with exposed muscle and red viable tissue in the pubis, lower abdomen, and right groin.

Wound culture with stains came back positive for polymicrobial gram-negative rods and gram-positive cocci. Two days after the procedure, the patient’s WBC count decreased to 30.9 x 10^9^/L, and the patient was afebrile. However, the right inguinal area was indurated, tender, and erythematous representing residual infection. The patient was taken to the operating room for another debridement. Intra-operatively, more necrotic tissue with purulent drainage was found in the perineum and inguinal area bilaterally. Re-wide debridement of skin, subcutaneous tissue, fascia, and muscle of 50 x 50 cm region was performed to remove the necrotic tissue completely. The patient tolerated the procedure well and was followed by an infectious disease specialist for empiric antibiotic treatment including ceftriaxone, vancomycin, and metronidazole. Her antibiotic regimen was tailored to vancomycin and ampicillin/sulbactam once the culture revealed Enterococcus faecalis. She was also followed by a wound specialist for a wound vacuum-assisted closure (VAC). After a week of intense wound care, intravenous antibiotic therapy, and glucose management, the patient was stabilized and discharged to an acute rehabilitation facility for further recovery. She was discharged on oral antibiotic therapy and a close follow-up plan in the outpatient clinic.

## Discussion

The rapid spread of necrotizing fasciitis on the integumentary system and all tissues below the skin lead to severe clinical manifestation and can be deadly if not contained in time. Though some people may present with different clinical signs, all victims of necrotizing fasciitis experience a rapid spread of the disease [6]. According to Chanemsetty et al., Fournier’s gangrene has the potential to spread at a rate of 2-3 cm per hour and is capable of causing multiple organ failure, hypotension, shock, and sepsis [7]. Despite the timely and effective medical interventions in the US, the condition leads to loss of limbs and sustaining life-long scars due to its rapid spread [8]. In every 10 people affected with necrotizing fasciitis who develop shock, six of them die, which attests to the complications leading to increased mortality [9]. Early diagnosis is important for proper containment of the condition, and any person who manifests with erythematous and edematous tissues should seek immediate clinical intervention to rule out necrotizing fasciitis.

The high mortality rate in necrotizing fasciitis is evident due to delayed diagnosis and poor management. Science-based evidence and empirical statistics reveal a mortality rate of 24% to 34% on average among affected patients [10]. Furthermore, the high mortality rate can be accounted for by underlying conditions such as trauma, obesity, DM, chronic renal failure, cirrhosis, immune deficiency, and peripheral vascular disease [10]. Alcoholism and smoking are behavioral predisposing factors for Fournier’s gangrene [11]. The lack of awareness of comorbidities negatively impacts the prognosis of affected patients. In the US, the number of obese people has been steadily increasing since the 1980s by a margin of 3% annually, and 35% of the population is said to be obese [12]. Obesity is a highly predisposing factor leading to necrotizing fasciitis [13]. Diabetic patients have an increased incidence of Fournier’s gangrene due to their immunosuppressive state, poor wound healing, defective phagocytosis, and existing small vessel disease [7]. According to Lira et al., diabetic patients with Fournier’s gangrene are found to have a higher mortality rate than non-diabetic patients at 71.9% versus 44.6% [11]. Poor management of diabetes significantly increases the susceptibility to infections, which further predisposes individuals to necrotizing soft tissue infection.

Male sex and age greater than 50 are non-modifiable risk factors that make individuals more prone to acquiring Fournier’s gangrene. Zhang et al. reported a male to female ratio of Fournier’s gangrene at 5.3:1. It is believed that the anatomical difference in the drainage of pelvic secretions between males and females is what decreases the susceptibility to Fournier’s gangrene in the female sex [14]. Patients suffering from acquired immunodeficiency syndrome, human immunodeficiency virus, autoimmune diseases, and those undergoing chemotherapy are extremely vulnerable to necrotizing fasciitis due to weakened immune systems [15]. 

Fournier’s gangrene treatment depends on the individual case and consists of emergent wide surgical debridement of nonviable and devitalized tissues, parenteral broad-spectrum antibiotic coverage, intense clinical hemodynamic support, and resuscitation with fluids [7,11]. Hyperbaric oxygen therapy has also been shown to benefit affected patients by stimulating collagen formation and enhancing leukocyte ability to kill aerobic bacteria [7,11]. Amputations can be indicated for management in severe shock and sepsis [10]. Empiric antibiotics are implemented immediately and altered once the specific microorganisms are identified. Antibiotic treatment must cover gram-negative bacteria, staphylococcal species, streptococcal species, coliforms, clostridium, bacteroides, and pseudomonas [7]. Indeed, an 80% mortality rate reported in necrotizing fasciitis cases has been infected by streptococcus [10]. Negative pressure wound therapy and wound VAC therapy are often used for treatment after surgical debridement. Wound VAC therapy helps increase blood supply leading to increased migration of inflammatory cells to the wound region, promoting healing and extended debridement [7]. Multiple surgical debridements are necessary to prevent future recurrence of Fournier’s gangrene. Patients who have had emergent surgical debridement of Fournier’s gangrene often require reconstructive surgery in the future. The outcome of the affected population improves with strict management of the comorbid conditions and fast medical decision-making for diagnosis and treatment in a timely manner.

Our patient returned to the hospital when the initial medical treatment failed to alleviate her symptoms. Her history of uncontrolled diabetes and obesity compounded with the genital abscess led to the rapid spread of the infection and emergent perineal debridement. While the patient needed multiple surgical procedures, causing an extended hospital stay, fortunately, she is recovering with intense medical treatment of broad-spectrum antibiotics and wound VAC therapy.

## Conclusions

Necrotizing fasciitis has a high mortality rate and is primarily seen in patients with comorbid conditions due to weakened immune systems. Fournier’s gangrene should be suspected in patients with a history of uncontrolled diabetes and urogenital infection. This case highlights the importance of prompt diagnosis and treatment of necrotizing soft tissue infection in a timely manner. While prompt diagnosis and treatment are necessary for the patient’s survival, the patient’s post-op recovery and wound healing are equally important. Multidisciplinary care is needed to ensure that the patient's comorbid conditions are well managed and patients are well educated on better management of their conditions and prevention of complications.
